# Novel Dormancy Mechanism of Castration Resistance in Bone Metastatic Prostate Cancer Organoids

**DOI:** 10.3390/ijms23063203

**Published:** 2022-03-16

**Authors:** Sanghee Lee, Theresa R. Mendoza, Danielle N. Burner, Michelle T. Muldong, Christina C. N. Wu, Catalina Arreola-Villanueva, Abril Zuniga, Olga Greenburg, William Y. Zhu, Jamillah Murtadha, Evodie Koutouan, Naomi Pineda, Hao Pham, Sung-Gu Kang, Hyun Tae Kim, Gabriel Pineda, Kathleen M. Lennon, Nicholas A. Cacalano, Catriona H. M. Jamieson, Christopher J. Kane, Anna A. Kulidjian, Terry Gaasterland, Christina A. M. Jamieson

**Affiliations:** 1 Department of Urology, University of California San Diego, La Jolla, CA 92093, USA; salee@health.ucsd.edu (S.L.); theresa.mendoza09@gmail.com (T.R.M.); danielleburner@gmail.com (D.N.B.); mmuldong@health.ucsd.edu (M.T.M.); catalinaarreola95@gmail.com (C.A.-V.); abrilz2296@gmail.com (A.Z.); miakicheva@gmail.com (O.G.); w.zhu@wustl.edu (W.Y.Z.); jmurtadh@ucsd.edu (J.M.); ekoutoua@ucsd.edu (E.K.); naomicorral.nc@gmail.com (N.P.); hpham0929@yahoo.com (H.P.); ckane@health.ucsd.edu (C.J.K.); 2 Moores Cancer Center, University of California San Diego, La Jolla, CA 92093, USA; c5wu@health.ucsd.edu (C.C.N.W.); cjamieson@health.ucsd.edu (C.H.M.J.); 3 Rady Children’s Hospital, San Diego, CA 92123, USA; 4 Department of Medicine, University of California San Diego, La Jolla, CA 92093, USA; gpineda@health.ucsd.edu (G.P.); klennon@health.ucsd.edu (K.M.L.); 5 Department of Urology, Korea University College of Medicine, Seongbuk-Gu, Seoul 02841, Korea; kkangsung76@korea.ac.kr; 6 Department of Urology, School of Medicine, Kyungpook National University, Daegu 41944, Korea; urohtkim@gmail.com; 7 Department of Radiation Oncology, University of California, Los Angeles, CA 90095, USA; ncacalan@ucla.edu; 8 Scripps MD Anderson Cancer Center, La Jolla, CA 92093, USA; kulidjian.anna@scrippshealth.org; 9 Scripps Institution of Oceanography, University of California San Diego, La Jolla, CA 92093, USA; tgaasterland@ucsd.edu; 10 Institute for Genomic Medicine, University of California San Diego, La Jolla, CA 92093, USA

**Keywords:** androgen pathway directed therapy, angiotensin-converting enzyme 2, basal-luminal-like hybrid, bone metastatic prostate cancer, dormant, enzalutamide, patient-derived organoids, patient-derived xenograft, prostate cancer San Diego 1, SARS-CoV-2, transmembrane protease serine 2

## Abstract

Advanced prostate cancer (PCa) patients with bone metastases are treated with androgen pathway directed therapy (APDT). However, this treatment invariably fails and the cancer becomes castration resistant. To elucidate resistance mechanisms and to provide a more predictive pre-clinical research platform reflecting tumor heterogeneity, we established organoids from a patient-derived xenograft (PDX) model of bone metastatic prostate cancer, PCSD1. APDT-resistant PDX-derived organoids (PDOs) emerged when cultured without androgen or with the anti-androgen, enzalutamide. Transcriptomics revealed up-regulation of neurogenic and steroidogenic genes and down-regulation of DNA repair, cell cycle, circadian pathways and the severe acute respiratory syndrome (SARS)-CoV-2 host viral entry factors, ACE2 and TMPRSS2. Time course analysis of the cell cycle in live cells revealed that enzalutamide induced a gradual transition into a reversible dormant state as shown here for the first time at the single cell level in the context of multi-cellular, 3D living organoids using the *Fucci2BL* fluorescent live cell cycle tracker system. We show here a new mechanism of castration resistance in which enzalutamide induced dormancy and novel basal-luminal-like cells in bone metastatic prostate cancer organoids. These PDX organoids can be used to develop therapies targeting dormant APDT-resistant cells and host factors required for SARS-CoV-2 viral entry.

## 1. Introduction

One in eight men will be diagnosed with prostate cancer (PCa), making it one of the leading health problems affecting today’s society [1]. Patients diagnosed during the earlier stages are surviving longer due to improved therapies and the prevalence of prostate-specific antigen (PSA) testing [1]. However, a growing number of these patients go on to develop advanced metastatic PCa [1]. In addition, since the 2012 USPSTF recommendation against PSA screening, there has been a step-wise increase in the number of men presenting at first diagnosis with higher grade, metastatic advanced prostate cancer [2]. The main standard-of-care treatment for advanced prostate cancer (PCa) is androgen pathway directed therapy (APDT) [1], also sometimes referred to as hormone therapy or androgen deprivation therapy (ADT) [3,4,5]. These treatments inhibit the activity of the male steroid hormone via the androgen signaling pathway, which normal prostate and most prostate cancers need to survive and proliferate [6]. APDTs can target the androgen receptor (AR) to competitively inhibit androgen binding and function, such as the anti-androgens bicalutamide, enzalutamide, apalutamide and darolutamide [6]. Other APDT therapies inhibit androgen hormone synthesis pathways, which produce testosterone and dihydrotestosterone (DHT), such as abiraterone which inhibits CYP17A1 [3,5]. Unfortunately, patients invariably become resistant to APDT, and develop castration-resistant prostate cancer (CRPC) [4,5,6]. Many mechanisms of CRPC have been uncovered, such as AR mutations, AR copy number amplification or loss, AR splice variants (ARVs), up-regulation of kinase pathways [7] that cross-talk with AR such as MAPKs or increase cell survival such as AKT/mTOR, along with mutations in Rb, p53 and in DNA repair genes such as BRCA2 [8]. Some of these mechanisms are being targeted in clinical trials; however, none are presently curative and the molecular mechanisms of AR-dependence and the transition to apparent AR-indifference of almost all prostate cancers under APDT pressure are still not understood [3,4,5,7]. 

Prostate cancer metastasizes to soft tissues such as lymph nodes, lung and liver but most often to bone [9,10,11]. Over 80% of advanced prostate cancer (PCa) patients develop bone metastases [1]. Standard-of-care APDTs, such as enzalutamide, can be helpful initially to prolong life; however, the majority of patients with bone metastases inevitably develop castration resistance [12,13]. As a result, many CRPC patients with bone metastases experience significant morbidity, including debilitating fractures and severe bone pain [14,15]. In addition, neuroendocrine PCa is emerging as a particularly aggressive, therapy-resistant metastatic PCa in patients treated with APDT [1,2,3,4,5,16]. There is an urgent need to develop new therapies for bone metastatic CRPC. 

Prostate tumors are highly heterogeneous [17]. Established prostate cancer cell lines have been used as cost-effective and readily available in vitro models of prostate cancer; however, they do not recapitulate the full complexity and heterogeneity of prostate tumors [18,19]. In addition, establishing models from prostate cancer patient tumors has been challenging because prostate cancer cells often become senescent upon isolation and usually do not grow in culture. Accordingly, there are relatively few prostate cancer cell lines and patient-derived xenograft (PDX) models of prostate cancer. Models of bone metastatic prostate cancer have been particularly challenging to establish due to the limited accessibility of patient bone metastases. We and others have been able to obtain rare tissue samples of fresh patient bone metastatic prostate cancer to establish patient-derived xenograft (PDX) models such as the some of the PDXs in the LuCaP and MDA series [16,20,21,22,23,24]. Through focused efforts, our PCSD series of PDXs were generated exclusively from orthopedic surgical bone metastatic prostate cancer patient specimens [20]. 

The establishment of three-dimensional cell cultures for bone metastatic prostate cancer have faced similar obstacles. 3D organoid culture systems developed by the Clevers group for a wide range of cancers allowed growth of reproducible cultures with niche-specific growth factors optimized for tumor cells from different tissues [25,26,27]. Under conditions optimized for prostate cancer, Karthaus and colleagues showed that prostate cancer organoids retained the histopathological and functional traits of their origin, including gene expression profile, androgen responsiveness, and drug resistance [25,28,29,30,31,32,33]. However, the number of bone metastatic PCa organoids remain limited. Here, we report the further optimization of conditions for the establishment of organoids from a patient bone metastatic prostate cancer xenograft model, PCSD1. Treatment with anti-androgen or androgen deprivation therapy led to the emergence of a novel dormant, castration-resistant sub-population in these prostate cancer bone metastasis organoids.

## 2. Results

### 2.1. Prostate Cancer PDX-Derived Organoids (PDOs) Recapitulate Heterogeneity and Androgen Pathway Directed Therapy Resistance of the Patient’s Tumor

**Figure 1:**
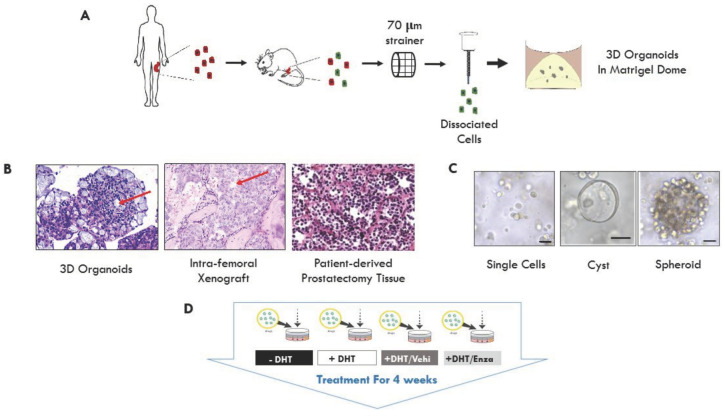

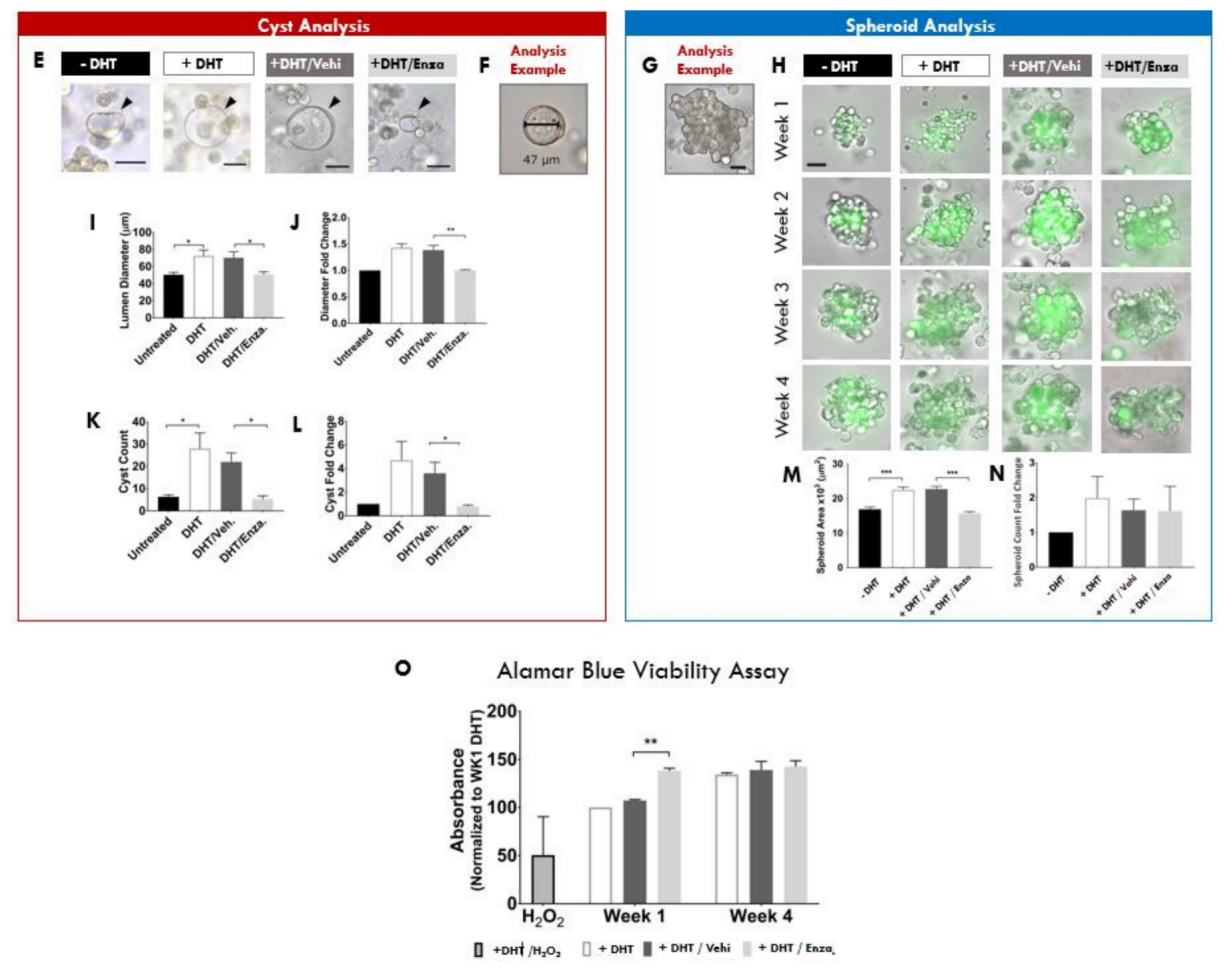
PDX-derived organoids (PDOs) recapitulate heterogeneity and androgen pathway directed therapy (APDT) resistance of the patient’s tumor. (**A**) Workflow to establish and optimize 3D organoids from patient-derived xenograft (PDX) model of prostate cancer bone metastasis. (**B**) Representative images of hematoxylin and eosin (HE) stained samples of prostatectomy tissue from the donor patient, PCSD1 intra-femoral xenografts and PCSD1 organoids. Red arrows point to structures seen in both the organoids and xenografts growing in the femur. HE images are shown at 10× magnification. (**C**) A heterogeneous mix of PCSD1 tumor cells grown in 3D organoid cultures consisting of single cells, hollow epithelial cysts and cell-filled spheroids. Bright-field microscope images of single cells and the spheroid are at 10× magnification and the cyst is at 20× magnification. Scale bars represent 50 µm. (**D**) Experimental design of 3D cultures of PCSD1-GFP/luciferase expressing organoids under four treatment conditions: -DHT, +DHT (1 nM), +DHT/Vehicle (1 nM/0.1% DMSO) and +DHT/enzalutamide (1 nM/10 µM). PCSD1 organoids in each treatment group were imaged weekly with a Keyence digital microscope for four weeks. (**E**) A representative image of cysts in each treatment condition after four weeks of treatment. (**F**) A measurement example of cyst lumen diameter using the Hybrid Cell Counter software. Scale bar for spheroid image is 50 µm. After measurement of cyst lumen diameter, the number of epithelial cysts greater than 50 µm in size was quantified in each culture. (**G**) An analysis example of spheroid area using the Hybrid Cell Counter software. Scale bar for spheroid image is 50 µm. (**H**) A representative image of spheroid in each treatment condition at week 1, 2, 3 and 4 of treatment. (**I**) Average lumen diameter of cysts. (**J**) Fold changes in lumen diameter. (**K**) Number of cyst count. (**L**) Fold changes in cyst count. (**M**) Average spheroid area. (**N**) Fold changes in the number of spheroids under four treatment conditions: −DHT, +DHT (1 nM), +DHT/Vehicle (1 nM/0.1% DMSO), and +DHT/enzalutamide (1 nM/10 µM). For fold change analysis in (**J**,**L**,**N**), quantified values were normalized to the DHT- condition. (**O**) The Alamar Blue viability assay performed on the cultures at weeks 1 and 4. Cell viability was determined using the 50 μL of the AlamarBlue reagent per well in 24-well plate (Invitrogen, by Life Technologies Inc., Carlsbad, CA, USA). Data represent the mean from four independent (*n* = 4) experiments performed in triplicate ± SEM. A student’s *t*-test was used to determine statistical significance (* indicate *p* < 0.05, ** indicates *p* < 0.01 *** indicated *p* < 0.0001).

Organoids were grown from intra-femoral PCSD1 patient-derived xenograft tumor cells. PCSD1 is one of the PDXs in the Prostate Cancer San Diego (PCSD) series, which we have established from surgical prostate cancer bone metastasis specimens (Figure 1A) [20]. The histomorphology observed in HE stained sections of PCSD1 organoids and xenografts (Figure 1B) was similar to that of the high-grade prostate adenocarcinoma Gleason grade 10 (5 + 5) seen in the donor patient’s prostatectomy specimen. Microscope images of live PCSD1 organoids showed that they contained a heterogeneous population of organoids, or “mini-tumors” made up of mostly of acinar cell-filled, “spheroids” and a smaller number of hollow epithelial “cysts” (Figure 1C). The PCSD1 organoids were treated with androgen pathway directed therapy (APDT) continuously for four weeks. APDT consisted of either: (a) no DHT being added to the PCa organoid culture media or (b) addition of the anti-androgen, enzalutamide. Specifically, the PCSD1 organoids were grown under the four treatment conditions: (1) no DHT (-DHT), (2) DHT (+DHT; +1 nM DHT), (3) Vehi (+DHT/Vehicle: +1 nM DHT/0.1% DMSO) and (4) Enza (+1 nM DHT/10 μM enzalutamide) as shown in Figure 1A,D. These cultures were maintained in the same wells and organoid media plus treatments were replaced every 3–4 days. 

The PCSD1 PDX organoids showed histologic morphology similar to the PCSD1 PDX grown in the mouse femur and to the original prostatectomy tissue of the donor patient’s tumor (Figure 1B). Prostate epithelial cells require androgens such as DHT to develop and function properly. Accordingly, the average cyst lumen diameter was significantly greater with DHT (Figure 1E–J) compared to no DHT (*p* < 0.05) and was significantly greater in DHT/Vehicle control than in DHT/Enza (*p* < 0.05). The total number of epithelial cysts was significantly increased (*p* < 0.05) plus DHT and decreased by 10 µM enzalutamide (*p* < 0.05) (Figure 1K–L). PCSD1 organoid cultures also contained spheroids, cell-filled clusters which made up the majority of the organoids (Figure 1G–N). The effects of APDT-resistance of the spheroids were visualized in time course microscope imaging of GFP in live organoids (Figure 1H). By week four, the average area, or size, of the spheroids had decreased in the cultures subjected to APDT, either no DHT (*p* < 0.0001) or plus enzalutamide (*p* < 0.0001). However, there was no significant difference in the total number of spheroids under any of the treatment conditions shown normalized to the total number of spheroids in no DHT in Figure 1N. In addition, Supplementary Figure S1 shows an example of 41 spheroids in a selected area of the Matrigel dome in one well of PCSD1 organoids from our standard culture protocol at a seeding density of 50,000 cells. Of note, the whole dome images of PCSD1 organoids in no DHT, DHT, DHT/Vehicle and DHT/Enza shown in Supplementary Figure S1 represent one focal plane in the z axis indicating robust formation of spheroids per well of PCSD1 organoids. APDT-resistance of the spheroids was confirmed in Alamar Blue viability assays, which showed that the PDX organoids were viable, thus resistant, after four weeks of continuous APDT (Figure 1O). These results show that both modalities of APDT selected a castration-resistant spheroid population in the PCSD1 organoids. 

### 2.2. Comparative Gene Expression Profiling of Androgen Pathway Directed Therapy (APDT) in PDX-Derived Organoids (PDOs)

**Figure 2:**
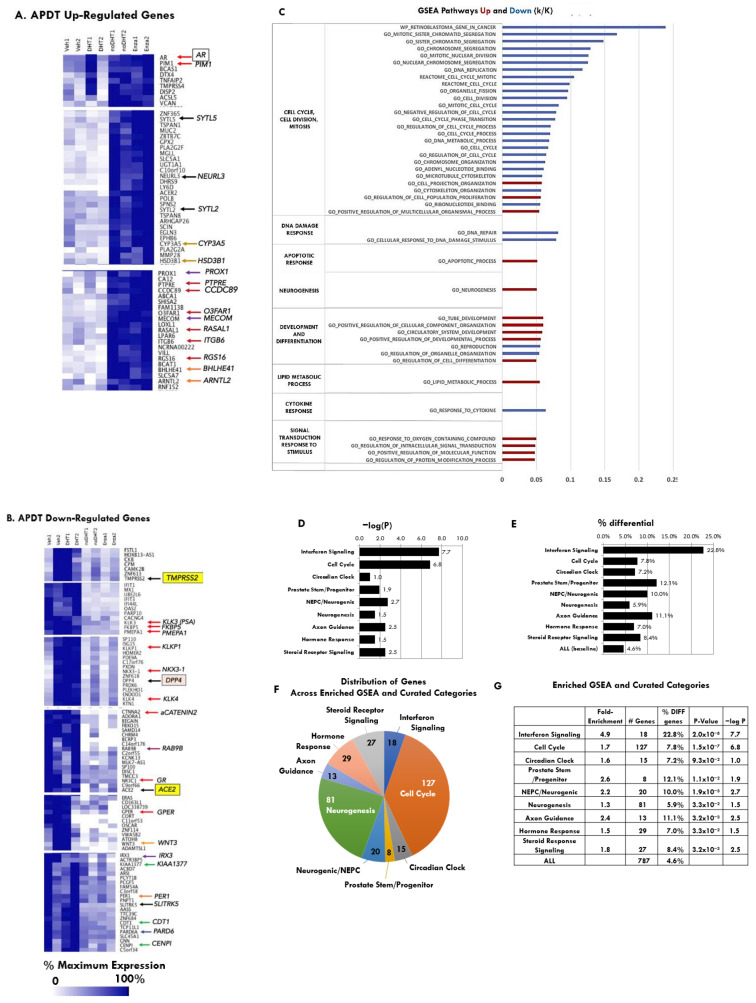
Gene expression profiling of APDT treated patient xenograft-derived organoids (PDOs) and gene set enrichment analysis (GSEA) of APDT up-regulated and down-regulated pathways in PDOs. (**A**) APDT significantly up-regulated genes, (**B**) APDT significantly down-regulated genes. (**C**) List of Gene Ontology (GO) pathways that were up-regulated (red) and down-regulated (blue) as evaluated using GSEA. Bars show the proportion (k) of total pathway genes (K), i.e., k/K values for GO annotation pathways which were up-regulated (red bars) or down-regulated (blue bars). (**D**) Bar graph showing−log(P) for each category of pathways, P calculated using Fisher’s exact test. (**E**) Bar graph showing percent of pathway genes with differential expression for each category. (**F**) Pie chart showing distribution of genes across enriched categories. (**G**) Enrichment scores and significant *p*-values for unified gene sets constructed from overlapping GSEA functional categories and literature.

To define the global gene expression landscape of the organoids under both modalities of APDT, we performed whole transcriptome RNA sequencing. The bioinformatics analysis strategy compared gene expression in organoids with AR agonism, that is, grown in plus DHT, or in Vehicle plus DHT, to those with AR antagonism, that is, no DHT or plus enzalutamide in two independent experiments. This strategy yielded an APDT-resistance signature of 787 differentially expressed genes: 312 genes down-regulated with high average expression (Down-Regulated Genes, High RPKM), 206 genes down-regulated with low average expression (Down-Regulated Genes, Low RPKM), 171 genes Up-Regulated Genes, High RPKM and 98 genes Up-Regulated Genes, Low RPKM with significant *p*-values. High/low average expression means high/low levels of normalized readcounts, and RPKM means reads per thousands bases per million mapped reads. Unsupervised hierarchical clustering was performed on the filtered gene lists of the four gene expression patterns (Figure 2 and Supplementary Figure S2). Heatmaps represent expression values for genes under the four treatment conditions in two independent experiments (Experiment 1 and Experiment 2) as described above: (1) Veh (+DHT/Vehicle: +1 nM DHT/0.1% DMSO), (2) DHT (+1 nM DHT), (3) no DHT (-DHT) and (4) Enza (+1 nM DHT/10 μM enzalutamide). 

Selected hierarchical clusters of genes with the same direction of expression change in both modalities of APDTs are shown in Figure 2A,B and Supplementary Figures S2 and S3. Gene expression data revealed that APDT significantly down-regulated the expression of known prostate and AR-target genes such as *TMPRSS2*, *KLK3(PSA)*, and significantly up-regulated genes involved in neuronal growth and synaptic function as well as enzymes involved in alternative androgen synthesis pathways such as *CYP3A5* and *HSD3B1*. Interestingly, *ACE2*, the host receptor for the SARS-CoV-2 “S” protein and DPP4, the MERS-CoV host receptor, were also significantly down-regulated by APDT. 

In addition to known AR target genes, APDT significantly down-regulated genes involved in cell cycle, cell division, mitosis and mitotic spindle structure and function (*AURKA*, *AURKB* and *CCNA2*), DNA synthesis and repair (*PAPRP9* and *BRCA1*), circadian clock gene PER1 and increased expression levels of two negative regulators of the circadian clock (*BHLHE41* and *ARNTL2*) (Figure 2A,B and Supplementary Figure S2). Notably, the glucocorticoid steroid hormone receptor, *GR*, which can reset the circadian clock, was also down-regulated. 

APDT significantly altered genes in the following signaling pathways: interferon signaling with down-regulation of *IRF7* and *STAT1*, which are involved in anti-viral response; WNT signaling with down-regulation of *WTIP*, *LBH* and *WNT3*, and up-regulation of *WNT4*; and epithelial to mesenchymal transition (EMT) with down-regulation of *PARD6* (Figure 2B). Interestingly, APDT led to up-regulation of genes involved in receptor tyrosine kinase (RTK) and serine/threonine kinase signaling and growth such as *EGFR*, *PIM1*, G-protein coupled receptor (*GPCR*) signaling (*RGS2*) and cytokine signaling (*TNFRSF21*, *TNFS8* and *TGFB3*). Many of these are actionable targets [34,35,36]. APDT led to significant up-regulation of genes involved in lipid metabolism, cholesterol and steroid hormone biosynthesis, including *HSD3B1* (Figure 2B and Supplementary Figure S2). Intriguingly, there was significant up-regulation of genes involved in neuronal development and synaptic function (*SEMA5A* and *SEMA3E*). Consistent with this was down-regulation of *SLITRK5*, an inhibitor of neuronal growth cone migration. Notably, APDT up-regulated *NRP1*, a pro-nociceptive factor and another SARS-CoV-2 receptor [37]. Finally, APDT led to up-regulation of developmental transcription factors involved in stem cells and cancer stem cells (*ETV4*, *SOX9*, *FOXO1* and *FOXO3*).

Comparisons of gene expression levels of 16 selected genes of interest under different androgen pathway directed treatments are shown as bar graphs of percentage of maximum RPKM (Supplementary Figure S3). APDT as either no DHT or enzalutamide treatment decreased expression of AR signature target genes, KLK3 (PSA), FKBP5 and TMPRSS2 as well as the MERS receptor, DPP4 and the SARS-CoV2 receptor, ACE2.

### 2.3. Gene Set Enrichment Analysis (GSEA) of APDT Up-Regulated and Down-Regulated Pathways in PDOs

Gene set enrichment analysis (GSEA) identified significantly down-regulated functional pathways, including interferon, cell cycle, cell division, mitosis, DNA damage response and cytokine response. GSEA also revealed significantly up-regulated functional pathways, including neurogenesis, development and differentiation, lipid metabolic process, partial apoptotic response and signal transduction response to stimulus (Figure 2C–G, Table 1 and Figure S2B). Enrichment ranged from 1.4-fold to 5.4-fold with *p*-values ranging from 0.032 (circadian clock) up to 2.6 × 10^−9^ (interferon signaling) and 5.8 × 10^−11^ (cell cycle) (Figure 2B–E; Supplementary Table S2). Therefore, gene expression clustering and GSEA revealed consistent changes in functional pathways in APDT-treated organoids. 

**Figure 3:**
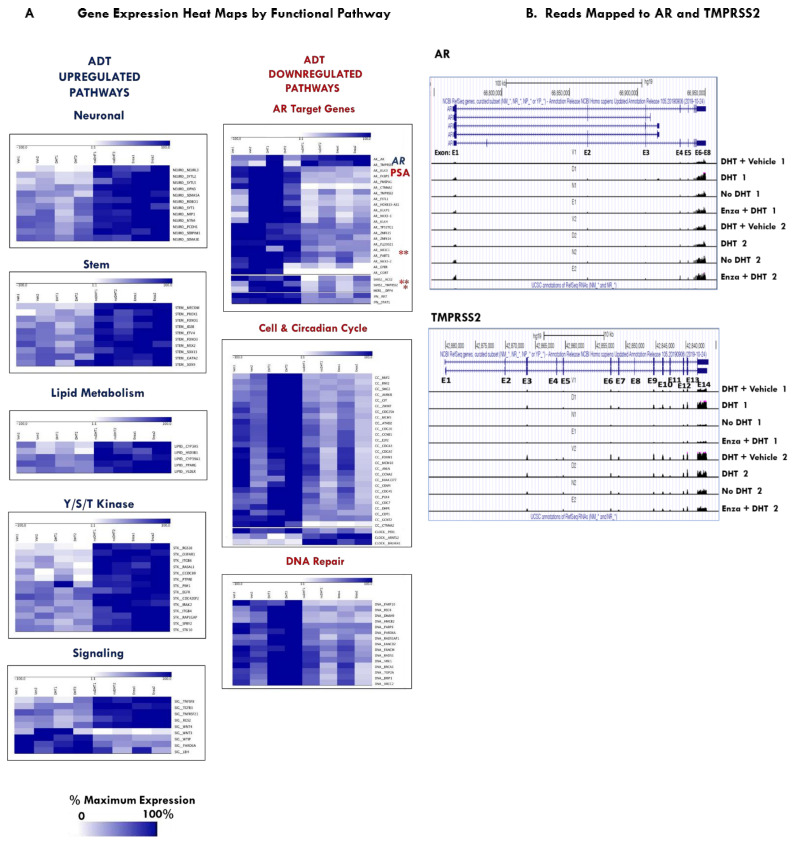
APDT up-regulated and down-regulated genes grouped by functional pathways. (**A**) Genes were clustered as percentage of maximum expression in functional classes and further classified according to the overall direction of gene expression change as either down-regulated or up-regulated. Gene expression is displayed as percentage of maximum normalized reads per kilobase of transcript per million mapped reads (RPKM). Color scale shows increasing % maximum expression as increasing intensity of blue indicates increasing mRNA expression level while increasing white represents decreasing % maximum expression and mRNA expression. (**B**) Genome aligned reads mapped to the AR gene in the top panel and the TMPRSS2 gene in the bottom panel. Alignments from top to bottom are Experiment 1: PCSD1 organoid samples treated with DHT+Vehicle, DHT, No DHT, enzalutamide plus DHT then Experiment 2: DHT+Vehicle, DHT, No DHT, enzalutamide plus DHT. AR gene shows accumulated reads under exons E1–E8, and for the TMPRSS2 gene: exons E1–E14.

Functional classes overrepresented in up-regulated and/or down-regulated genes in Figure 2 were ranked by the percent of differential genes in the class and further classified according to the overall direction of gene expression change. Hierarchical clustering heat maps of the specific genes were grouped by their GSEA and curated functional pathways. Functional classes with genes consistently up-regulated included neuronal, stem, lipid metabolism, kinases and signaling pathways, including TNFα, WNT and TGFβ (Figure 3A). Functional classes with genes consistently down-regulated under APDT included AR-regulated target genes, cell cycle, circadian cycle and DNA repair genes (Figure 3A).

Reads mapped to the human reference genome (GRCh37/hg19) were analyzed for read depth for each exon across the AR and TMPRSS2 gene regions (Figure 3B). Alternative splicing in AR and gene fusion events for TMPRSS2 with ETS-family members are involved in prostate cancer. A prognostic biomarker of CRPC and mechanism of APDT-resistance, is the ARV7 alternatively spliced variant of AR. ARV7 lacks exons 5–7, which delete the androgen-binding domain, thus, converting AR into a constitutively active isoform. The aligned reads for AR do not show a read depth bias that would indicate mRNA transcripts that lack exons 5–7 (Figure 3B, top panel). However, there is an increase in the AR exon reads in ENZA+DHT1 from Experiment 1 relative to VEH+DHT1 and in ENZA+DHT2 relative to VEH+DHT2, no DHT2 and +DHT2 from Experiment 2 consistent with the increased in AR mRNA RPKM expression levels. There were no reads that indicated a fusion event for TMPRSS2, which is consistent with previous RT-PCR analyses showing that PCSD1 PDX tumors do not have the TMPRSS2-ERG fusion [20]. The aligned reads show decreased read depth in the APDT samples for TMPRSS2 consistent with the decrease in RPKM, hence expression level, which is especially evident at the 3′ exons (Figure 3B, bottom panel).

### 2.4. The Anti-Androgen, Enzalutamide, Decreased the Protein Levels of AR and TMPRSS2 in APDT-Resistant PDO 

**Figure 4:**
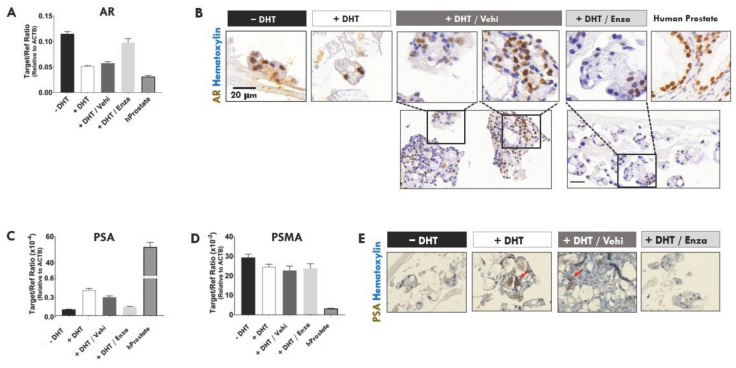

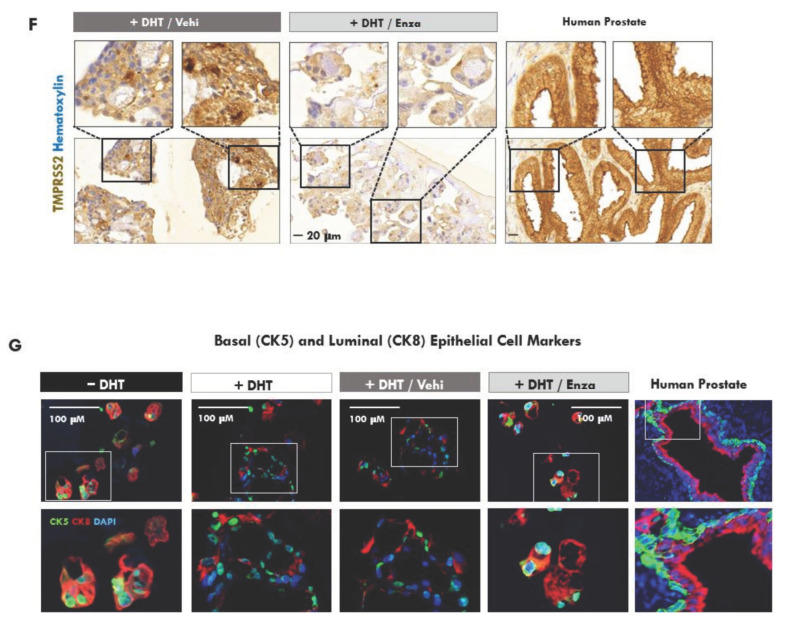
The anti-androgen, enzalutamide, decreased the protein levels of AR and TMPRSS2 in ADT-resistant PDO. (**A**) Quantitative RT-PCR analysis for AR level in PCSD1 3D organoids under the four treatment conditions: −DHT, +DHT (1 nM), +DHT/Vehicle (1 nM/0.1% DMSO) and +DHT/enzalutamide (1 nM/10 µM). Human beta actin was used as the internal control, reference gene (delta delta C_t_ target/reference). (**B**) IHC analysis of AR expression in PCSD1 3D organoids. Representative digital microscope images are shown of AR IHC staining performed on 4% paraformaldehyde fixed, paraffin embedded 5 µm sections from four culture conditions. Human prostate tissue was used as a positive control. (**C**) Quantitative RT-PCR analysis for PSA level in PCSD1 3D organoids. (**D**) Quantitative RT-PCR analysis for PSMA level in PCSD1 3D organoids under the four treatment conditions. Graphs represent one experiment performed in duplicate. (**E**) Immuno-histochemical (IHC) analysis of PSA expression in PCSD1 3D organoids. Red arrows point to PSA positive staining. (**F**) IHC analysis of TMPRSS2 expression in PCSD1 3D organoids. Representative digital microscope images are shown of (**B**,**E**,**F**) IHC staining performed on 4% paraformaldehyde fixed, paraffin embedded 5 μm sections from four culture conditions. Data represent the mean from four independent (*n* = 3) experiments performed in triplicate. (**G**) Confocal FV1000 images of IFC performed on PCSD1 3D organoids and normal human prostate cancer control tissue to visualize the prostate epithelial cell markers: cytokeratin 8 (CK8), a luminal epithelial cell protein, and cytokeratin 5 (CK5), a basal epithelial cell protein along with DAPI nuclear stain. Top five panels: PCSD1 3D organoids and patient prostate cancer tissue showed CK8 protein (red), and CK5 protein (green). DAPI stained the nuclei (blue). Inset (white square) shows further magnified image of organoids in panels 1–4 and normal prostate in panel 5 with CK8 (red) and CK5 (green) immunostaining. In the PCSD1 organoids, immunofluorescent cytochemical (IFC) analysis of CK5 and CK8 revealed heterogeneous expression patterns. Confocal microscope imaging of the control tissue, normal human prostate, showed the expected pattern of CK5^+^ basal cells and CK8^+^ luminal cells in glandular structure.

In organoids without DHT or with enzalutamide, AR mRNA levels increased as seen in RNA sequencing (Figure 2A) and confirmed in quantitative RT-PCR assays (Figure 4A). Conversely, AR protein levels decreased in the enzalutamide-treated organoids as shown using anti-AR IHC (Figure 4B and Supplementary Figure S6A). The effects of androgen deprivation on PSA, a transcriptional target of AR, and PSMA were evaluated. Androgen deprivation (no DHT or +DHT/Enza) reduced PSA mRNA expression and PSA protein (Figure 4C–E). The relatively low PSA and high PSMA mRNA expression in the organoids were consistent with the levels in the donor patient and PCSD1 PDX [20,21]. PSA protein expression was heterogeneous in PCSD1 organoids as was seen previously in PCSD1 PDX [21]. Androgen deprivation (+DHT/Enza) significantly reduced TMPRSS2 protein expression (Figure 4F) as seen in transcriptomics (Figure 2A, Figure 3, Figure 4B and Supplementary Figures S2, S3 and S5). Analysis of prostate luminal and basal cell markers in PCSD1 organoids showed that APDT produced mostly CK5^+^CK8^+^ cells with large CK5^+^ nuclei and CK8^+^ in the cytoplasm (Figure 4G), which were also seen in PCSD1 intra-femoral xenograft tumors (Supplementary Figure S6B). Thus, both modalities of APDT, either no DHT or plus enzalutamide, induced a novel population of CK5^+^CK8^+^ basal-luminal-like hybrid cells in the organoids.

### 2.5. Androgen Pathway Directed Therapy Induced Dormancy in Castration-Resistant PDOs 

**Figure 5:**
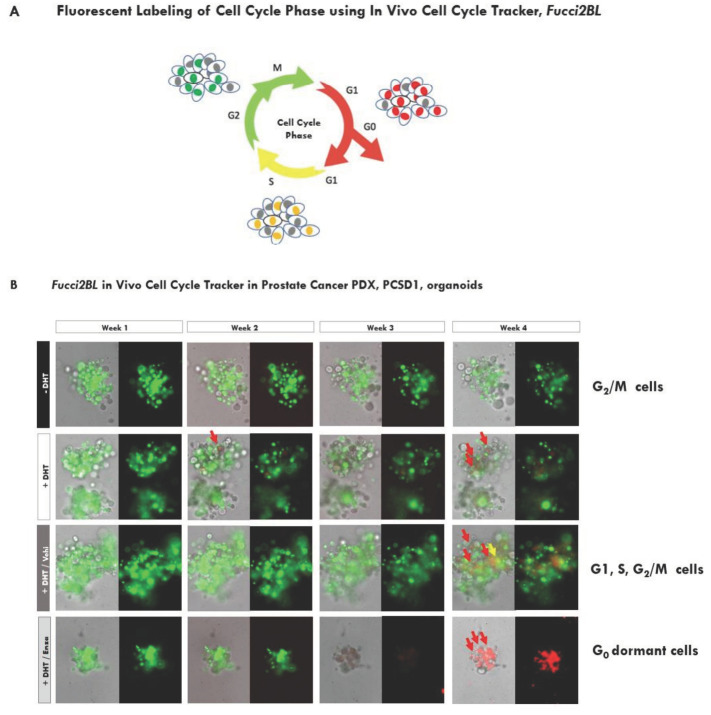

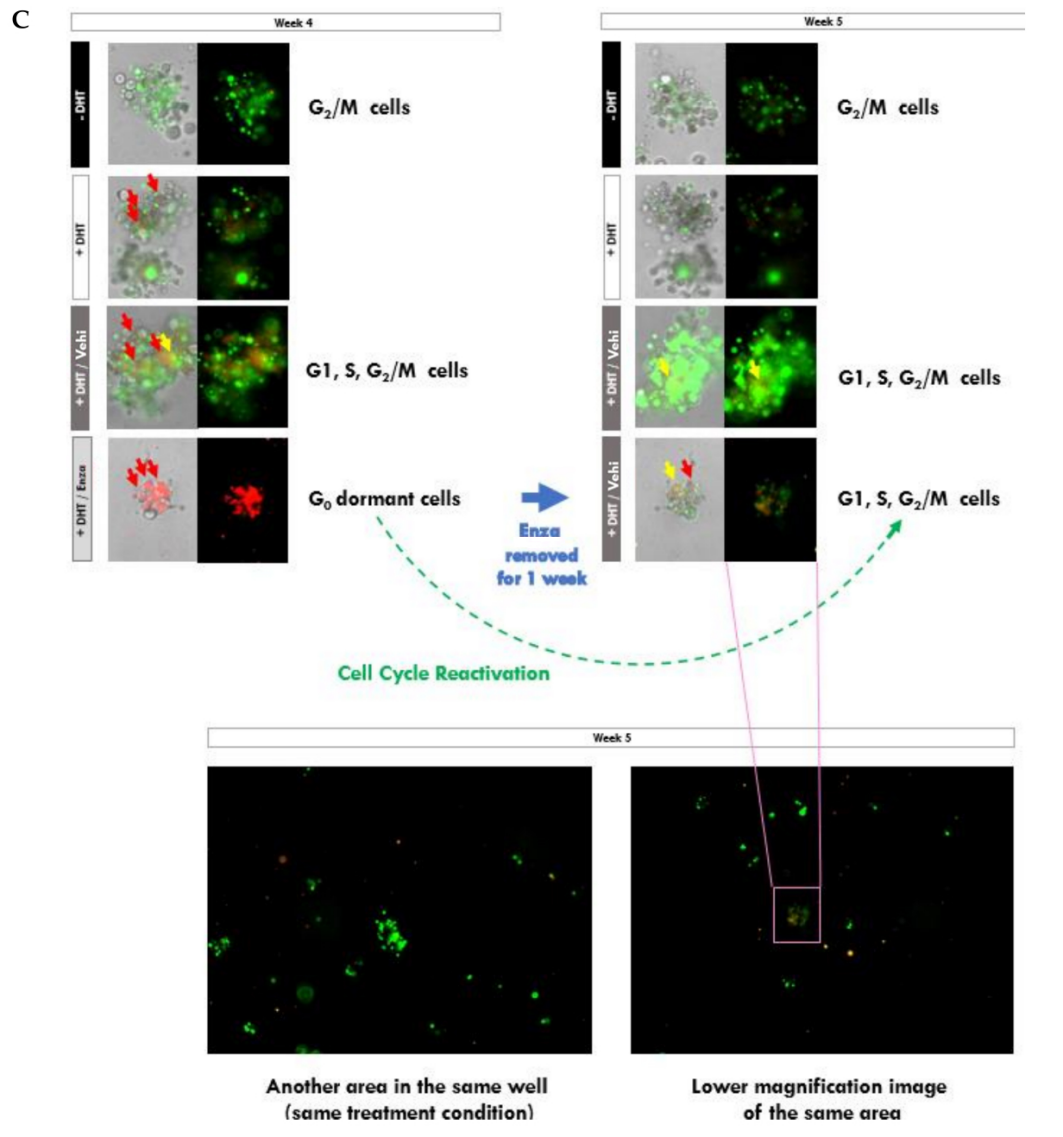
Androgen pathway directed therapy induced a novel population of dormant cells in PDX organoids. (**A**) The *Fucci2BL* fluorescent, ubiquitination-based cell cycle indicator reporter system visualizes the phase of cell cycle shown by colorimetric signal of red, yellow and green fluorescence for G_1_, G_1_/S and S/G_2_/M, respectively. (**B**) Time course of cell cycle stages in live PCSD1 organoids under the four treatment conditions. Representative images for the same organoids followed through time in each treatment condition at week 1, 2, 3, and 4 of treatment of PCSD1 organoids stably expressing the *Fucci2 BL* bicistronic fluorescent, ubiquitination-based cell cycle indicator reporter system showing three cell cycle phases: G_1_/G_0_ by red fluorescence, G_1_/S by yellow fluorescence and G_2_/M by green fluorescence. The images of bright-field, red fluorescent channel and green fluorescent channel were obtained and merged. (**C**) Removal of enzalutamide led to cell cycle reactivation in dormant PCSD1 organoids. +DHT/enzalutamide containing media was removed from PCSD1 organoids after 4 weeks of treatment and replaced with +DHT/Vehicle media. Microscope images at 4 weeks prior to media change and at 5 weeks after 1 week of removal of enzalutamide containing media are shown for the same organoid.

To determine the status of the cell cycle in the live organoids over time, we used the live cell cycle tracker system, *Fucci2BL* (Figure 5A–C). In the Fucci2BL system the ubiquitin regulated cell cycle sensor, mCherry-hCDT (red fluorescence), is expressed in cells in the G1 or G0 phases of the cell cycle and mVenus-hGEM (green fluorescence) is expressed in G2/M cells (Figure 5A) [38]. The cell cycle sensors, or probes, overlap in expression in S phase (yellow fluorescence). By 4 weeks, the majority of PCSD1 organoids in +DHT/Enza shifted to the bright red fluorescent G_0_ phase while the organoids in +DHT/Vehicle contained cells in the green fluorescent G_2_/M phase, yellow S phase or red G1 phase, indicating they were cycling (Figure 5B). Enzalutamide/+DHT containing media was then removed from PCSD1 organoids after 4 weeks of treatment and replaced with Vehicle/+DHT media. Microscope images taken 1 week after of removal of enzalutamide media and replacement with Vehicle/+DHT media for a total of 5 weeks of growth in the same wells, showed that cells expressed red, green and yellow fluorescent sensors indicating they had reactivated the cell cycle as shown in Figure 5C and Supplementary Figure S4. Therefore, removal of enzalutamide reversed exit from the cell cycle to G_0_ showing that the inactivity of the cell cycle was temporary. These results, taken together with the gene expression profiling results showing down-regulation of cell cycle genes, are consistent with the live Enza-treated PCSD1 cells shifting to a reversible G_0_ phase over time and thus, becoming dormant, castration-resistant bone metastatic prostate cancer cells.

## 3. Discussion

**Figure 6:**
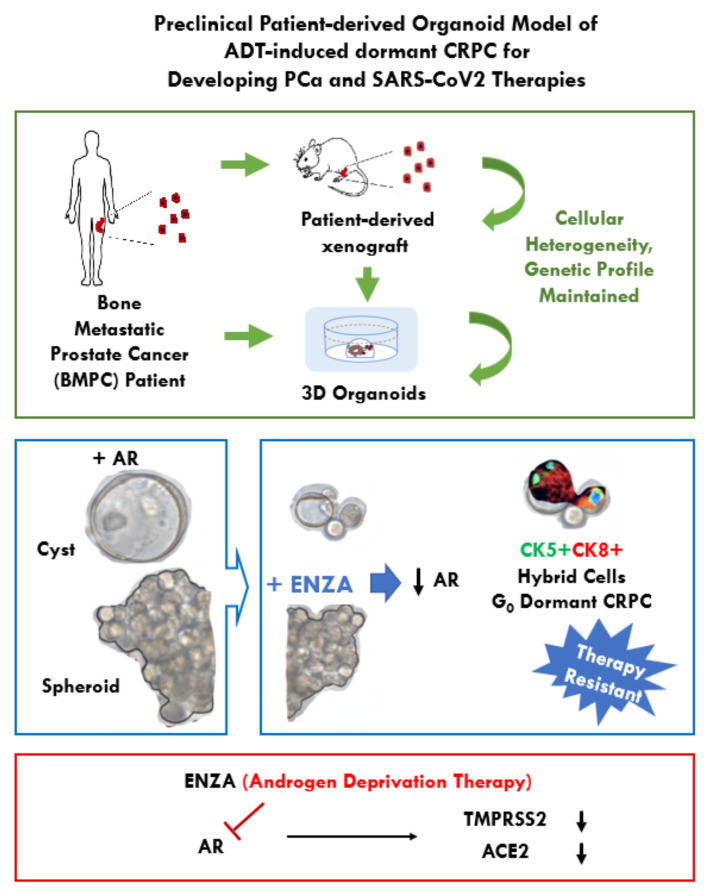
Novel dormancy mechanism of castration resistance in a bone metastatic prostate cancer organoid model. Overview of the PCSD1 patient-xenograft derived organoid (PDO) model for bone metastatic prostate cancer and its response to androgen pathway directed therapy. This model can be used for further understanding of tumor resistance, progression and dormancy under therapies that suppress the androgen pathway.

In this study, we established a new bone metastatic prostate cancer organoid model using tumor cells from the patient-derived prostate cancer bone metastasis xenograft, PCSD1, and investigated their response to androgen pathway directed therapies (APDT). Castration-resistant PDX-derived organoids (PDOs) emerged when they were treated with either of the two main strategies for APDT: depriving the tumor of androgen, that is, no DHT, or direct binding inhibition of the androgen receptor (AR) with the anti-androgen, enzalutamide. Time course analysis of the cell cycle revealed that enzalutamide induced a gradual transition into dormancy as shown here in real time at the single cell level while maintaining the 3D multi-cellular context of the living organoids using the *Fucci2BL* fluorescent live cell cycle tracker system. The enzalutamide-induced exit from the cell cycle into G_0_ was reversible in the same cells upon removal of enzalutamide from the culture. Furthermore, we identified a corresponding dormancy-associated gene expression profile, which was similar to dormancy-associated gene expression profiles seen by Morrissey and colleagues in disseminated tumor cells (DTCs) from prostate cancer patient bone marrow biopsies as well in other cancer models of dormancy [39,40,41]. The emergence of dormant tumor cells with a unique basal-luminal-like phenotype in this PDX organoid model represents a novel mechanism of castration resistance in bone metastatic prostate cancer under APDT therapy. The dormancy CRPC gene signature identified in this study may be useful for identifying new targets to eradicate dormant bone metastatic CRPC before further progression and recurrence of a more recalcitrant disease (Figure 6). 

Transcriptomic analysis of the PDOs revealed that APDT up-regulated neurogenic, stem, kinase and steroidogenic genes and, conversely, down-regulated well-known prostate and AR target genes, interferon signaling, DNA repair, cell cycle, mitosis, cell division and circadian cycle genes. Interestingly, APDT decreased the expression of ACE2 and TMPRSS2, the SARS-CoV-1 and SARS-CoV-2 host viral entry receptor and co-factor, respectively, and the MERS receptor, DPP4, in the CRPC organoids. Mapped reads to exons analysis of expression levels confirmed the gene expression changes for AR and TMPRSS2 and further revealed that ARV splice variants were not detected and TMPRSS2 did not form a fusion gene with ERG in the PCSD1 organoids. Immunohistochemical analysis further showed that enzalutamide decreased the protein levels of AR as well as TMPRSS2 in the PDOs.

The main advantage of organoid models is that they retain the features of the tissue of origin, including heterogeneity of the tumor, the stem cells, their progenitors and differentiated cells [25,26,27,30,32,42,43,44,45,46]. This has been a challenge for bone metastatic prostate cancer due to the rarity of fresh prostate cancer bone metastasis specimens and the difficulty of establishing prostate cancer organoids compared to other cancers. This organoid study complements other prostate organoid studies looking at higher throughput approaches for treatment combinations that may be effective, but we sought to more specifically look at the response itself since inducing cell death and cell cycle arrest are common endpoints for drug efficacy screens using organoid cultures. The Fucci2BL live cell cycle tracking system in these unique prostate cancer bone metastasis organoids revealed not only that enzalutamide induced dormancy but also that the length of the cell cycle was very long even in the presence of androgen receptor function. In the organoids with androgen signaling, that is +DHT or +DHT/Vehicle, we observed a long pause in G2/M from week 1 to week 3 with the transition into G1 and S phases occurring at 4 weeks. This may be due to the lack of signaling pathways present in the unique bone environment, which are not present under the conditions in this study. Several studies have shown that the bone microenvironment provides essential WNT, TGFbeta, Notch and FGF pathway signaling [47,48,49,50]. Nagaya and co-workers identified higher expression of the CXC chemokine signaling genes, including CXCL1, CXCL3, CXCL6, CXCR1 and CXCR2 in prostate cancer bone metastases [51]. Indeed, we have shown previously that the PCSD1 PDX cells grew significantly faster in the bone niche in vivo as intra-femoral xenografts compared to sub-cutaneous xenografts ([20,21] and Muldong et al., manuscript in preparation). Future studies will aim to better match the unique and complex bone niche such as co-culturing the organoids with bone marrow-derived stoma, osteoclasts and osteoblasts. Dondossola and colleagues have developed an engineered bone mimetic environment for organoids and showed their response to Radium 223 therapy closely mimicked the response in the in vivo bone environment [52]. Future studies with the PCSD1/Fucci organoids will be aimed at systematically adding in the bone niche signaling and supporting bone niche cells and bone matrix to more closely recreate the bone microenvironment.

Distal metastases can remain dormant or quiescent for months or even decades, then recur as a rapidly growing, metastatic and therapy-resistant lethal disease. Dormant cells transiently down-regulate cell cycle machinery and exit the cell cycle into G_0_ to become quiescent while maintaining proliferative capabilities. This is well-studied in hematopoietic stem cells and shown to occur in lymphoid malignancies in the bone marrow niche where cancer stem cells can become quiescent, and thus, attain therapy resistance [53,54]. Dormant disseminated tumor cells (DTCs) of bone metastatic prostate cancer have been identified in PCa patient bone marrow biopsies [55]. Single cell RNA-sequencing analysis revealed that the DTCs were highly heterogeneous within and between patients and demonstrated considerable plasticity in prostate cancer markers [39,40]. Morrissey and colleagues identified single cell gene expression profiles and signatures of dormant DTCs involving a p38α/β regulated quiescence transcription factor network that contained proliferation/growth arrest and pluripotency/self-renewal genes reminiscent of the APDT-down-regulated pathways seen in this study [40]. Interestingly, the DTC signature of 26 up-regulated genes included BHLH4E1, a negative regulator of the circadian cycle, which we also identified here as a significant APDT up-regulated gene [39,40]. Intriguingly many of the APDT-induced changes in the PDX organoids were similar to the embryonic diapause like (EDL) expression profile of very early stage mouse embryos that have paused in development due to stressful conditions, which was recently shown to overlap with the expression profiles described for chemo-resistant dormant, treatment-persistent breast cancer cells [40]. Changes included down-regulation of processes related to metabolism, post-transcriptional RNA processing and transport, protein synthesis and proliferation, including DNA replication and chromosome segregation while processes related to extracellular matrix (ECM) reorganization, such as collagen modification and cell adhesion, including integrin binding, were up-regulated [41]. Thus, the PDOs help to fill an urgent need for accurate patient-derived model systems to study dormancy and therapy resistance in bone metastatic prostate cancer.

Several features of the 3D multi-cellular PCSD1 organoids were consistent with the PDX and the patient of origin in addition to histology [20,21]. First, PSA expression was heterogeneous and low in PCSD1 organoids as seen in the PDX tumor and in the patient; second, PCSD1 organoids expressed GFP and luciferase as in the PDX; third, the PCSD1 organoids expressed AR mRNA which increased in the CRPC organoids under APDT as seen previously in the PDX [21]; fourth, the organoids expressed the prostate basal cell marker, CK5, and the prostate luminal cell marker, CK8, similar to the PDX; fifth, whole genome RNA sequencing showed that APDT decreased the expression of well-known AR-target genes prostate-specific genes such as KLK3 (PSA) and NKX3.1. Therefore, the PCSD1 organoids arose from the purified PDX tumor and recapitulated characteristics and responses of the PDX in vivo and the patient [21]. 

The PDX organoid cultures were heterogeneous and made up of cysts and spheroids, which responded differently to APDT. Spheroids were APDT-resistant, thus castration resistant, and contained basal-luminal-like (CK5+CK8+) hybrid cells. Cysts on the other hand, were a minor, castration (APDT)-sensitive population. Epithelial cysts and spheroids have also been seen in 3D cultures from other epithelial tissues such as breast, lung, and colon [27,56]. Breast cancer cells, which had less malignant properties, tended to form epithelial cysts with a hollow lumen while more malignant cells tended to form cell-filled acini reminiscent of their growth as tumors in patients [56]. Similarly, the prostate PDX-organoid cysts studied here may be more differentiated and less malignant while the spheroids may be the more malignant tumor sub-population.

The novel APDT-resistant, dormant CK5^+^CK8^+^ hybrid cells identified in organoids here may be similar to transition states or intermediates seen in prostate development or inflammation. In previous studies, intermediate prostate cells, which are CK5^+^CK18^+^ or CK5^+^CK8^+^, have been observed in human prostate inflammatory atrophy (PIA) [57] and mouse models of prostate cancer [58]. Recently, PIA-like, low CD38-expressing, inflammation-associated luminal cells were shown to initiate prostate cancer [59]. Such intermediates, or transition states, are found in other cancers as well. A hybrid epithelial to mesenchymal (E/M) transition state was highly tumorigenic in breast cancer cell lines. This transition state may lie in a spectrum of lineage plasticity states normally involved in the regenerative response to damage and co-opted for tumorigenesis [60,61]. In PCSD1 organoids, APDT treatment produced CK5^+^CK8^+^ hybrid epithelial cells, which may be similar to intermediate cells. The newly identified basal-luminal-like hybrid prostate cancer cells may represent a type of APDT-induced stasis, that is, a castration-resistant dormant state poised for re-current growth. Further characterization of CK5^+^CK8+ basal-luminal-like hybrid cells will assess expression of additional basal epithelial markers, p63 and CK14, to determine if these cells are p63^-^CK5^+^CK8+CK14+ transitional progenitor cells or a novel treatment emergent cell in bone metastatic prostate cancer.

The *Fucci2BL* live cell cycle tracker system allowed us for the first time to follow the time course of APDT-induced changes in the cell cycle in the same cells while maintaining critical cell–cell interactions in the 3D organoid structure. Tomura et al. developed transgenic mice with the *Fucci* (fluorescent ubiquitination-based cell cycle indicator) probes to understand the factors involved in cell cycle transition and dormancy in lymphoid cells in vivo and to track these changes at the single cell level in real time [38]. They showed by FACS analysis of DNA content (DAPI) and proliferation (Ki67) that the ubiquitin regulated cell cycle sensor, mCherry-hCDT, accurately tracked the G1 and G0 cells, and mVenus-hGEM tracked with the G2/M cells [38]. Pineda et al. used these probes to develop a bicistronic lentiviral-based system, *Fucci2BL*, to transduce primary human cells from normal and patient chronic phase myelogenous leukemic (CP CML) CD34+progenitor cells [62]. Using DAPI/Ki67 FACS, they validated the high fidelity of the *Fucci2BL* system for quantification of cell cycle kinetics in human cell lines and primary cells. Here, we introduced the *Fucci2BL* lentiviral system into PCSD1 PDX cells and established xenografts and organoids to determine the cell cycle changes due to androgen pathway directed therapies at the single cell level in the context of the live 3D organoids. We found that after one week in the presence of DHT, the organoids were in a prolonged G2/M mVenus-hGEM green fluorescent phase and at four weeks, the spheroids contained cycling cells in G1, S and G2/M. This indicates that the length of cell cycle of the PDX organoids was very long and the cells were triggered to divide at 4 weeks. In the presence of enzalutamide, the organoids were also in the G2/M phase at one and two weeks; however, at three weeks they began to lose green fluorescence and at four weeks showed strong mCherry-hCDT red fluorescence, an indicator of G_0_. Gene expression changes were also consistent with the G_0_ dormant state since RNASeq expression profiling of the enzalutamide-treated organoids showed down-regulation of cell cycle, cell division and DNA repair pathways. Strikingly, removal of enzalutamide and replacement with DHT/Vehicle reversed the G0 arrest and the cells re-entered the cell cycle. Thus, the PCSD1 PDX-organoids showed enzalutamide-inducible dormancy, defined as a temporary state of inactivity or hibernation, and the corresponding gene expression profiles were similar to dormancy-associated gene expression profiles seen in DTCs from prostate cancer patient bone marrow biopsies and in other cancer models of dormancy [39,40,41].

Investigation of AR expression showed that enzalutamide-treatment led to a decrease in AR protein even though there was an increase AR mRNA levels, as shown by RNAseq and quantitative RT-PCR. Mapped reads analysis showed that AR splice variants (ARVs), which delete C-terminal exons, were not detected. Immunohistochemical analysis of AR was performed with an antibody specific for the N-terminal region of AR protein so loss of AR protein levels in IHC analysis indicated that ARV proteins were not significantly expressed. The slow growing organoid cultures did not yield sufficient protein for western blot analysis. But now that we have established optimized conditions for growing the PDX organoids and identified the key time points in our time course experiments. Our next goal is to determine the appropriate protocol for scaling-up the organoid cultures for further studies of the mechanism. 

A similar APDT response has been previously described in which AR mRNA was expressed and de novo AR protein synthesis was continuous in prostate stem cells treated in the APDT, flutamide; however, steady-state AR protein levels were undetectable due to continuous degradation via the E3 ligase, MDM2 and proteasome [63]. The lack of steady-state AR protein was required to maintain an AR negative, prostate cancer stem cell phenotype [63]. AR protein degradation with long-term enzalutamide treatment is likely linked to the mechanism of castration-resistance in the PCSD1 organoids. 

In this study, we showed that APDT was sufficient to down-regulate the expression of both TMPRSS2 RNA and protein and the RNA level of ACE2, in the PCSD1 organoids. Recently, the AR-inducible gene, transmembrane protease serine 2 (TMPRSS2), was identified as a critical host viral entry factor for severe acute respiratory syndrome coronavirus 2 (SARS-CoV-2), the virus that causes coronavirus disease 2019 (COVID-19). SARS-CoV-2 infects cells using its Spike protein (S), which binds to its host receptor, angiotensin-converting enzyme 2 (ACE2), on host cells [64]. TMPRSS2 was shown to be required for proteolytic processing the S protein once it is bound to ACE2 to allow fusion of the virus with the cell [64]. TMPRSS2 has been intensively studied in prostate cancer as it is an AR-up-regulated gene, which occurs as a gene fusion of its promoter with ETS family transcription factors in over two-thirds of prostate cancer patients [10,11,19]. 

Androgens have recently been shown to regulate ACE2 and TMPRSS2 in lung organoids as well [17]. Androgen regulation of ACE2 has been implicated in studies showing that male alveolar lung cells have higher expression of AR, TMPRSS2 and ACE2 compared to female cells that anti-androgens can inhibit SARS-CoV-2 infection in vitro [65]. It has been observed in studies from multiple COVID-19 pandemic epicenters, including Wuhan, China, Veneto, Italy and New York, USA, that men have a worse COVID-19 disease course and higher mortality compared to women [66,67]. The increased COVID-19 disease severity and death rate in men may be due in part to androgen induction of increased TMPRSS2 and ACE2 expression in men. As was reported recently in an immunocompromised patient with chronic lymphocytic leukemia (CLL) [68], prostate cancer patients who have very high TMPRSS2 in their cancer may be especially vulnerable and may constantly shed the virus. Interestingly, prostate cancer patients on APDT in Veneto, Italy who became infected with COVID-19 had fewer hospitalizations and a shorter disease course than prostate cancer patients not on APDT [69]. Some subsequent studies have shown no effect of APDT on COVID-19 infection or disease severity in PCa patients, so this remains an open question [55]. There are currently ongoing clinical trials to determine whether APDT drugs may be repurposed as a short-term early treatment to reduce TMPRSS2 and ACE2 expression and thus inhibit SARS-CoV-2 virus levels in patients for whom immune-based therapies, such as vaccines or antibodies, are ineffective or unavailable [65,70,71,72,73]. This PCSD1 PDX-organoid model is a potential model for testing therapeutic interventions that target viral infections. Future studies will further validate this model for drug development to determine if TMPRSS2 and ACE2 down-regulation in this model may also down-regulate viral entry of SARS-CoV2, for example, using pseudo-type luciferase reporter viruses. 

We showed here for the first time that castration-resistant bone metastatic prostate cancer organoids treated with enzalutamide exited from the cell cycle to a reversible G_0_ state, that is, a temporary state of inactivity or hibernation known as dormancy and identified a dormancy-associated gene signature. This is, therefore, a unique patient-derived 3D ex vivo organoid model for deeper understanding of tumor resistance, progression and dormancy as well as for testing novel therapies for castration-resistant bone metastatic prostate cancer.

## 4. Materials and Methods

### 4.1. Patient-Derived Xenograft Model and 3D Organoid Culture

This study was carried out in strict accordance with the approval by the University of California San Diego (UCSD) Institutional Review Board (IRB). Informed consent was obtained from all human participants. Biospecimens were collected by the Moores Cancer Center Biorepository and Tissue Technology Shared Resource from consenting patients under a University of California, San Diego Human Research Protections Program Institutional Review Board approved protocol (HRPP No. 181755CX). A surgical prostate cancer bone metastasis de-identified specimen was harvested from a patient who progressed to castrate resistant bone metastatic prostate cancer, which we called Prostate Cancer San Diego 1 (PCSD1). Intra-femoral injection of PCSD1 was performed to establish a patient-derived xenograft (PDX) model [20,21,74]. Briefly, cells from PCSD1 surgical specimen were resuspended in high concentration Matrigel (BD Biosciences, Inc., Franklin Lakes, NJ, USA) at a 1:1 ratio and injected intra-femorally (IF) into the right femur of 6 to 8-week-old male *Rag2^−/−^;γ_c_^−/−^* mice. PCSD1 cells were freshly isolated from intra-femoral xenograft tumors and transduced with a GFP-luciferase expressing lentiviral vector (GLF), a kind gift from Dr. Catriona Jamieson, UCSD, as previously described. PCSD1_GFP_luciferase cells were then sorted by flow cytometry for GFP positive cells on FACSAria prior to being intra-femorally injected into male *Rag2^−/−^;γ_c_^−/−^* mice. All animal protocols were performed under a UCSD animal welfare IACUC approved protocol.

### 4.2. Organoid Growth

PCSD1 cells were maintained as intra-femoral tumors in male *Rag2^−/−^;γ_c_^−/−^* mice prior to establishing 3D cultures. On the day of harvest, tumors were removed immediately after sacrificing and submersed in cold DMEM/F-12 complete media (DMEM/F-12 supplemented with 10% (*v*/*v*) FBS and 1% (*v*/*v*) penicillin streptomycin). Tumors were processed with some modifications to our previously established methods [20]. Briefly, tumor samples were minced to 1–3 mm^3^ sized pieces and digested with 10 mL Accumax Cell Dissociation solution (Stem Cell Technologies, Vancouver, Canada) for 45 min at room temperature. Digestions were neutralized with the addition of 10 mL DMEM/F-12 complete. The suspension was filtered through a 70 µm cell strainer (BD Biosciences, Franklin Lakes, NJ, USA), centrifuged at 1200 RPM for 5 min at 4 °C, and the cell pellet was washed three times with fresh DMEM/F-12 complete media. A magnetic mouse cell depletion kit (Miltenyi Biotec catalog no. 130-104-694, Bergisch Gladbach, Germany) was used to enrich for human cells in our PDX tumor samples. Final cell counts were determined using 1:1 dilution in trypan blue dye and counted on a hemocytometer. PCSD1 tumor cells were embedded in a dome of growth factor reduced Matrigel (BD Biosciences) at a seeding density of 50,000 cells per 40 µL of Matrigel. 3D organoid cultures grown in prostate organoid medium [26] with the addition of fetal bovine serum. DHT and enzalutamide were added to the medium at a final concentration of 1 nM and 10 µM, respectively. The vehicle control was 0.1% (*v*/*v*) DMSO in culture medium. The epithelial cyst lumen diameter, cyst counts, spheroid area and spheroid counts were determined using a Keyence BZ-X710 microscope (Keyence Corporation, Osaka, Japan). 

One main advantage of maintaining tumor cells in a three-dimensional matrix is that the resulting cell populations retain their ability to respond to signaling factors and drugs in a way that is similar to their clinical response [26,30]. Aliquots of the cell suspension were domed on cell culture plates according to the seeding concentration as previously described [26]. To prevent cell adhesion to the bottom of the plate, tissue culture plates were inverted prior to being placed in the CO_2_ incubator (5% CO_2_, 37 °C) for 15 min. Once the Matrigel domes solidified, the appropriate volume of medium, as previously described, was added to each well. Media formulations were modified from Drost et al. [26]. To improve the establishment and long-term viability of PCSD1 3D cultures, fetal bovine serum (FBS) was added to the culture medium. With this modified medium, the PCSD1 3D cultures remained viable in vitro for several weeks. An additional modification was the separation of human cells from mouse cells in PDX tumor samples using a mouse cell depletion magnetic beads column. After magnetic separation, human cell enriched populations contained a negligible number of mouse cells and cell viability was not significantly reduced during this process. The medium was changed every 3 to 4 days and fresh medium was made every week. After 7 days, cultures were grown in medium without the Rho-kinase inhibitor Y-27632. 

To passage the organoids, media was aspirated and the Matrigel domes were rinsed with cold passaging media (advanced DMEM/F12 containing 5% FBS, 1X penicillin/streptomycin, 10 mM HEPES, and 2 mM GlutaMAX) and then collected into conical tubes using a cell scraper. The organoids were mechanically dissociated with a 1.0 mL syringe attached to a 25 G needle. Once dissociated, 10 mL of cold passaging media was added, and the solution was centrifuged at 1200 RPM for 5 min at 4 °C. The resulting cell pellet was incubated with 2 mL 1X TrypLE Express (ThermoFisher, Waltham, MA, United States) in a 37 °C incubator with gentle shaking to further dissociate the cultures into single cell suspensions. The TrypLE Express enzyme was inactivated through the addition of 10 mL of passaging media and the solution was centrifuged at 1200 RPM for 5 min at 4 °C. The resulting cell pellet was resuspended in growth factor reduced Matrigel (BD Biosciences) and domed on cell culture plates, as previously described [26].

### 4.3. Cyst and Spheroid Analysis

For functional analysis, we divided the observed phenotypes in our cultures into two main categories: epithelial cysts and spheroids. However, a common limitation in utilizing heterogeneous in vitro models is the challenge of reproducible, and robust quantitation which takes into account the inter- and intra-tumor heterogeneity. We developed new quantitation methods for the cysts and spheroids, which allowed us to measure the size and number of cysts and spheroids using microscope image analysis under different treatment conditions. The epithelial cyst lumen diameter, cyst counts, spheroid area, and spheroid counts were observed using a Keyence BZ-X710 microscope (Keyence Corporation). Lumen diameter was measured with an adjustable scale bar using the Keyence microscope software (Figure 2B). Spheroid area was measured using the Keyence Hybrid Cell Counter at 4× magnification by outlining spheroid clusters that were at least 50 μm in size with the Free Draw Tool (Figure 2B).

### 4.4. RNA Sequencing Analysis and Bioinformatics

RNA sequencing was performed on bulk RNA from the organoids extracted using a Qiagen RNEasy micro extraction kit and sequenced with 100 base-pair paired end reads (PE100) to a depth of approximately 25 million reads per sample on an Illumina NovaSeq 6000. Total RNA was assessed for quality using an Agilent Tapestation 4200, and samples with an RNA integrity number (RIN) greater than 7.9 were used to generate RNA sequencing libraries using the TruSeq Stranded mRNA sample prep kit with TruSeq Unique Dual Indexes (Illumina, San Diego, CA, USA). Samples were processed and resulting libraries were multiplexed and sequenced with 100 basepair (bp) paired end reads (PE100) to a depth of approximately 25 million reads per sample on an Illumina NovaSeq 6000. Samples were demultiplexed using bcl2fastq v2.20 Conversion Software (Illumina, San Diego, CA, USA). Extracted RNA was sequenced and evaluated to generate raw and normalized read counts and normalized for each treated organoid. 

The goal of the RNA sequencing was to identify genes responsive to APDT through analysis strategies that could take into account the heterogeneity across organoid cultures. Recognizing that each organoid potentially represented a different clone within the cancer, a query-based analysis method was developed to accommodate for inter-organoid variability. Genes were first filtered by consistent direction of response across organoid experiments (1 and 2), and then ranked by degree of fold change and consistency of their response to the androgen deprivation treatments. 

Our analysis strategy allowed us to compare each set of organoids subjected to the same treatments, ranked the genes inversely by variability within same treatments, and then generated a global ranking of genes based on variability and degree of fold change. The gene expression pattern of organoids in growth media with no DHT was expected to correlate with AR inhibition with enzalutamide; growth media with DHT was expected to correlate with vehicle. To derive normalized read counts (RPKM, reads per thousand mRNA bases, per million reads mapped), reads were mapped to the human genome (GRCh37/hg19, February 2009; March 2020 update) using Bowtie2 (v2.1.0), duplicate reads removed, and read counts generated for 23,137 publicly annotated genes with Cufflinks (v1.3.0) [75,76]. Raw read counts were derived as an integrity check using samtools (v1.4) on indexed bam files and UCSC hg19 genome coordinates for annotated genes (10 January 2020, update) [77]. Genes with no mapped reads were set aside leaving 17,004 genes for further evaluation. Each group was submitted to gene set enrichment analysis (GSEA, v4.1.0 with MSigDB 7.2) [78] to identify functional categories of genes enriched among and across the four groups of genes. *p*-values for these fold-change enrichment scores were calculated using Fisher’s 2 × 2 exact test (FET).

GSEA evaluates enrichment for sets of genes (gene sets) derived from multiple databases and from many experiments with data deposited in public, curated repositories and reported in the literature [47,48,49,79,80,81,82,83,84,85,86,87,88,89,90,91,92,93]. Since many gene sets overlap and share a functional category theme, overlapping gene sets were downloaded from GSEA and merged to create curated sets of genes for functional categories with highly differential key regulatory genes: (1) Interferon Signaling (102 genes), (2) Cell Cycle (2038 genes), (3) Circadian Clock (240 genes), (4) Neurogenesis (1701 genes), (5) Axon Guidance (129 genes; evaluated as a subset of Neurogenesis), (6) Hormone Response (529 genes; including glucocorticoid response), and (7) Steroid Receptor Signaling (385 genes). The Interferon Signaling merged gene set is the union of genes in GSEA’s GO_RESPONSE_TO_TYPE1_INTERFERON, and REACTOME_INTERFERON_ALPHA_BETA_SIGNALING. The Cell Cycle merged gene set unifies GO_CELL_CYCLE, REACTOME_CELL_CYCLE, and GO_REGULATION_OF_CELL_CYCLE. The Circadian Clock merged gene set unifies GO_CIRCADIAN_REGULATION_OF_GENE_EXPRESSION, REACTOME_CIRCADIAN_CLOCK, PID_CIRCADIAN_PATHWAY, GO_REGULATION_OF_CIRCADIAN_RHYTHM, BIOCARTA_CIRCADIAN_PATHWAY, GO_PHOTOPERIODISM, GO_ENTRAINMENT_OF_CIRCADIAN_CLOCK, GO_CIRCADIAN_RHYTHM. The Neurogenesis merged gene set unifies GO_NEUROGENESIS, GO_NEURON_DEVELOPMENT, GO_NEURON_DIFFERENTIATION, and KEGG_AXON_GUIDANCE. The KEGG Axon Guidance gene set was evaluated separately as a particularly enriched subset of Neurogenesis. The Steroid Receptor Signaling merged gene set unifies GO_CELLULAR_RESPONSE_TO_STEROID_HORMONE_STIMULUS and GO_RESPONSE_TO_STEROID_HORMONE.). Two additional lists of genes were derived through literature review: (8) Prostate Stem/Progenitor (96 genes) and (9) Neuro-Endocrine Prostate Cancer (NEPC)/Neurogenic (269 genes). Enrichment scores were calculated for each merged functional category using the ratio of the percent of differential genes in the category compared to the percent of differential genes overall. (Overall, 4% of 17,004 genes were identified as differentially expressed using the filtering queries described above.) *p*-values for these fold-change enrichment scores were calculated using Fishers 2 × 2 Exact Test (FET).

### 4.5. PCSD1 Organoid Spin Down Method 

The PCSD1 organoids were harvested and processed using a spin down method as previously described [42]. Briefly, the PCSD1 cells were incubated with 1 mL of Cell Recovery solution for 60 min at 4 °C after removal of culture media. For cell collection, the Cell Recovery solution was aspirated and then 1 mL of cold PBS was added to 1.5 mL tube, after which the domes were mechanically crushed with a P1000 pipet and transferred to a 1 mL Eppendorf tube. The cell suspension was centrifuged at 1200 RPM, 4 °C. PCSD1 pellets were fixed in 500 µL of 4% paraformaldehyde (PFA) for 60 min at room temperature (RT) and resuspended in 200 µL of warm agarose (2% in H_2_O). Solidified agarose pellets were carefully dislodged from the Eppendorf tube using a 25 G needle and transferred to 70% ethanol. Dehydration and paraffin embedding were processed using standard protocols and 5 μm sections were cut on microtome. 

### 4.6. Quantitative RT-PCR

RNA was extracted from PCSD1 organoid cultures using Qiagen RNeasy kit and, RNA was quantified using a NanoDrop. cDNA was synthesized using Superscript III (Invitrogen, by Life Technologies Inc., Carlsbad, CA, United States) and used for quantitative PCR using Light Cycler 480 SYBR-Green I Master kit (Roche Inc., Basel, Switzerland) as previously described in Godebu et al., 2014 [21]. Custom-designed human-specific primers were used for AR, prostate specific antigen (PSA) and prostate specific membrane antigen (PSMA) as previously described [20]. Human and mouse-specific ACTB-specific primers were used as an internal reference control for qPCR [21]. Positive control RNA included human prostate RNA (Clontech, Mountain View, CA, United States).

### 4.7. Immunohistochemisty (IHC) and Immunofluorescent Chemistry (IFC) 

Heat-induced antigen retrieval was performed using Leica Bond Epitope Retrieval Buffer 2 (EDTA solution, pH 9.0) for 20 min. Endogenous peroxidase was blocked using BloxAll for 20 min. IHC analyses were performed using a rabbit monoclonal antibody against N-terminal residues of human AR (cell signaling Cat No. 5153S, RRID: AB_10691711), a rabbit Poly-HRP-IgG secondary antibody Novolink polymer against rabbit (Leica, Cat No. RE7280-CE, lot No. 6058461) and 3′3-diaminobenzidine (DAB; brown). A hematoxylin nuclear counterstain (blue) was applied. IFC were performed using a chicken polyclonal antibody against CK5 (BioLegend Covance Cat No. PRB-160P-100, RRID: AB_291581), a mouse monoclonal antibody against CK8 (BioLegend Covance Cat No: MMS-162P-250, RRID: AB_291334), a goat Alexa 488 antibody against chicken, Alexa 594 and Alexa 647 (all Alexa antibodies, Thermo Fisher).

### 4.8. Cell Cycle Imaging Using Lentiviral Bicistronic Fluorescent, Ubiquitination-Based Cell Cycle Indicator Reporter (Fucci2 BL) System

The cell cycle phase of the PCSD1 organoids were determined by Lentiviral Bicistronic Fluorescent, Ubiquitination-based Cell Cycle Indicator Reporter (*Fucci2 BL*) System, which is designed to visualize the cell cycle of G_1_ by red fluorescence, G_1_/S by yellow fluorescence, and G_2_/M by green fluorescence. The *Fucci2 BL* expression vector was designed and generated to have mVenus-hGem(1/110) and mCherry-hCdt1(30/120) in Pcdh-T2A-copGFP (CD521A-1, SBI Systems Biosciences) as previously reported [62]. The PCSD1 cells were transduced with a multiplicity of infection (MOI) of 200 *Fucci2 BL* and maintained for three weeks for live cell fluorescent microscopic imaging using the Keyence BZX 710. The images of bright-field, red fluorescent channel and green fluorescent channel were obtained and merged. 

### 4.9. Statistical Analysis 

Data from cyst/spheroid analysis and enzalutamide dose titration of epithelial cyst response represent the mean of three independent (*n* = 4) experiments performed in triplicate ± SEM. Data from quantitative RT-PCR of AR, PSA and PSMA represent the mean of two experiments performed in triplicate ± SEM. Data from viability assay represent the mean of two experiment performed in triplicate ± SEM. Statistical significance (* *p* < 0.05, ** *p* < 0.01, **** *p* < 0.0001) for all the experiments was determined using the Student’s *t*-test.

## 5. Conclusions

Androgen pathway directed treatment of organoids from a patient-derived xenograft of bone metastatic prostate cancer led to dormant, castration-resistant prostate cancer cells with novel characteristics. Dormancy was reversible upon removal of the anti-androgen, enzalutamide. The APDT-induced dormancy-associated gene expression profiles showed down-regulation of cell cycle, cell division, DNA repair and circadian cycle pathways with up-regulation of stem-cell transcription factors, steroidogenic and neurogenic pathways reminiscent of those seen in bone metastasis prostate cancer DTCs, chemo-resistant persister cells and the embryonic diapause-like (EDL) signature in dormant, chemo-resistant breast cancer organoids. In addition, APDT decreased levels of the SARS-CoV-2 host cell viral entry factors, TMPRSS2 and ACE2, thus, the PDX organoids may be used to test therapies that decrease viral entry of SARS-CoV-2 and its variants for immunocompromised patients in whom vaccines may not be protective. The bone metastatic prostate cancer PDX organoids can be used as models to develop therapies that target the APDT-induced dormancy signature in order to eradicate dormant CRPC cells.

## Figures and Tables

**Table 1:** Selected genes of interest in APDT-signature from PDX PCSD1 organoids.

Direction of Gene Expression Change with APDT	Category	Selected Genes of Interest
Down	Prostate and AR target genes	*TMPRSS2*, *KLK3* (*PSA*), *KLK4*, *KLKP1*, *FKBP5*, *PMEPA1*, *NKX3-1*, *TP53TG1*, *ZNF614*, *ZNF615*, *PART1*, *FLJ20021*, *GR*, *GPER*, *NKX3*-2
	Cell cycle, cell division, mitosis and miotic spindle structure and function	*NUF2*, *RMI2*, *SMC2*, *AURKA*, *AURKB*, *CCNA2*, *ZWINT*, *MCM5* *ATAD2*, *CDC20*, *CCNB1*, *E2F2*, *CDC3A*, *CDC25A*, *KIA1377* *CDT1*, *CENPI*, *GCNT2*, *DHFR*, *CDC45*, *CIT*, *PLK4*, *CDC7*
	DNA synthesis and repair	*HMGB2*, *PAPRP9*, *BRCA1*, *FANCD2*, *BRIP1*, *XRCC2*, *FANCM*, *VRK1*, *PAD51*, *REC8*, *DNAH9*, *RAD51AP1*
	Circadian clock	*PER1*
	Interferon signaling	*IRF7*, *STAT1*
	WNT signaling	*WTIP*, *LBH*, *WNT3*
	Epithelial to mesenchymal transition (EMT)	*PARD6*
	SARS-CoV2 host viral entry factors	*TMPRSS2*, *ACE2*
Up	Serine/threonine kinase signaling and growth	*EGFR*, *PIM1*, *RAPGAP1*, *SPRY2*, *CDC42EP2*, *PTPRE*, *CCDC89*, *O3FAR1*, *RASAL1*, *ITGB6*, *RGS16*
	G-protein coupled receptor (GPCR) signaling	*RGS2*
	Cytokine signaling	*TNFRSF21*, *TNFS8*, *TGFB3*
	Circadian clock inhibition	*BHLHE41*, *ARNTL2*
	Lipid metabolism, cholesterol, and steroid hormone biosynthesis	*CYP3A5*, *HSD3B1*, *VLDLR*, *PPARG*, *CYP39A1*
	Neuronal function and development	*NRP1*, *ROBO1*, *SYT1*, *NTN4*, *PCDH1*, *SERPINI1*, *SYTL5*, *SYTL2*, *SEMA5A*, *OPN3*, *SEMA3E*
	Developmental transcription factors in stem cells and cancer stem cells	*ETV4*, *SOX13*, *MSX2*, *SOX9*, *FOXO1*, *FOXO3*, *PROX1*, *MECOM*, *ID2B*, *GATA2*

## Data Availability

All data analyzed during this study are included in this published article and its supplementary materials files. Raw data are available upon request from the corresponding author and are not publicly available due to ethical restrictions.

## Supplementary Materials

The following are available online at https://www.mdpi.com/article/10.3390/ijms23063203/s1.
